# Supplementary material for: State-of-the-art violence detection techniques in video surveillance security systems: a systematic review
Source: PeerJ Comput Sci. 2022 Apr 6;8:e920. doi: 10.7717/peerj-cs.920 (PMC9044356; doi:10.7717/peerj-cs.920)
Supplement: Supplemental Information 1 [file peerj-cs-08-920-s001.pdf]

**State-of-The-Art Violence Detection Techniques in Video Surveillance Security Systems: A Systematic Review: Systematic Literature Review Protocol**

The purpose of the study described in this protocol is to review the current status of State-of-the-art violence detection techniques in video surveillance security systems since 2015 to 2021 using a tertiary study to review articles related to it in particular articles describing Systematic Literature reviews (SLRs).

Research questions

| **ID** | **Research question** | **Motivation** |
|---|---|---|
| RQ1 | What kind of video based violence detection techniques are applied in state-of-the-art researches? | Identify state-of-the-art methods and techniques in intelligent video surveillance |
| RQ2 | What kind of video features and descriptors are used in video-violence detection? | Identify commonly used and state-of-the-art features and descriptors in video violence detection |
| RQ3 | What datasets are used to train models for video-violence detection | Identify datasets commonly used in intelligent video surveillance |
| RQ4 | What challenges and open questions exist to identify violence in videos? | Identify challenges and open issues in intelligent video surveillance |

Search Process

The search process is a manual search of journal papers in specific sources between January, 2015 to December, 2021. The nominated sources are shown in the following Table.

**Sources to be Searched**

| **#** | **Source** | **Link** |
|---|---|---|
| 1 | Science Direct | sciencedirect.com |
| 2 | IEEE Xplore | ieeexplore.ieee.org |
| 3 | Springer | link.springer.com |
| 4 | Wiley | onlinelibrary.wiley.com |
| 5 | Scopus | scopus.com |
| 6 | Conference on Computer Vision and Pattern Recognition (CVPR) | Sources of CVPR conference between 2015 to 2021 |
| 7 | The International Conference on Computer Vision (ICCV) | Sources of ICCV conference between 2015 to 2021 |
| 8 | European Conference on Computer Vision (ECCV) | Sources of ECCV conference between 2015 to 2021 |

**Inclusion criteria**

- Articles on the following topics those belong to journal papers and conference papers that published in CVPR, ICCV, ECCV conferences, published between January 1st 2015 and December 31st 2021, will be included

- Review or survey i.e. Literature surveys with defined research questions, search process, data extraction and data presentation
- Research articles - Articles aim to solve some specific research problems using deep learning methods, and gives some theoretical propositions, solutions, and provides laboratory experiments or industrial implementations.

**Exclusion Criteria**

The following types of papers will be excluded:
Duplicated papers – The same paper that appears multiple times.
Non-research papers – The paper is not a research article. It can be Editorial notes, comments, etc.
Non-related papers – The topic under study goes beyond the research context of this work.
Non-English papers – The paper is not written in English.
Implicitly related papers – The paper does not directly express the research focus on video surveillance security systems.
Non-research papers – The paper is not a research paper. It can be editorial notes, comments, etc.

Primary study selection process

The results will be tabulated as follows:

- Number of papers per year per source
- Number of candidate papers per year per source
- Number of selected papers per year per source.

The relevant candidate and selected studies will be selected. The rejected studies will be checked later. We will maintain a list candidate papers that were rejected with reasons for the rejection.

**Quality Assessment**

Each article will be evaluated using the York University, Centre for Reviews and Dissemination (CDR) Database of Abstracts of Reviews of Effects (DARE) criteria (http://www.york.ac.uk/inst/crd/crddatabase.htm#DARE). The criteria are based on four questions:

- Are the review's inclusion and exclusion criteria described and appropriate?
- Is the literature search likely to have covered all relevant studies?
- Did the reviewers assess the quality/validity of the included studies?
- Were the basic data/studies adequately described?

**The questions are scored as follows:**

Question 1: Y (yes), the inclusion criteria are explicitly defined in the paper, P (Partly), the inclusion criteria are implicit; N (no), the inclusion criteria are not defined and cannot be readily inferred.

Question 2: Y, the authors have either searched 4 or more digital libraries and included additional search strategies or identified and referenced all journals addressing the topic of interest; P, the authors have searched 3 or 4 digital libraries with no extra search strategies or searched a defined but restricted set of journals and conference proceedings; N, the authors have search up to 2 digital libraries or an extremely restricted set of journals.

Question 3: Y, the authors have explicitly defined quality criteria and extracted them from each primary study; P, the research question involves quality issues that are addressed by the study; N no explicit quality assessment of individual papers has been attempted.

Question 4: Y Information is presented about each paper; P only summary information is presented about individual papers; N the results of the individual studies are not specified.

The scoring procedure is Y=1, P=0.5 and N or Unknown=0. The data will be extracted by one researcher and checked by another.

Data Collection

The data extracted from each paper will be:

The source (i.e. the conference or journal).

The year when the paper was published. Note if the paper was published in several difference sources both dates will be recorded, and the first date will be used in any analysis. This is necessary in order to track the activity over time.

Classification of paper

Type (Literature Review or Survey (S), Research paper (RP))

Scope (Research trends or specific research question).

Main software engineering topic area.

The author(s) and affiliation (organization and country).

Research question/issue.

The number of primary studies used in the S/RP

Summary of paper.

Quality score for the study.

The extracted will be checked by tutor.

Data Analysis

The data will be tabulated (ordered alphabetically by the first author's name) to show the basic information about each study. The number of studies in each major category will be counted. The tables will be reviewed to answer the research questions and identify any interesting trends or limitations in current video-based violence detection research as follows:

Question 1: What kind of video based violence detection techniques are applied in state-of-the-art researches?

Motivation: Identify state-of-the-art methods and techniques in intelligent video surveillance.

Question 2: What kind of video features and descriptors are used in video-violence detection?

Motivation: Identify commonly used and state-of-the-art features and descriptors in video violence detection.

Question 3: What datasets are used to train models for video-violence detection?

Motivation: Identify datasets commonly used in intelligent video surveillance.

Question 4: What challenges and open questions exist to identify violence in videos?

Motivation: Identify challenges and open issues in intelligent video surveillance.
